# Mycobacterium tuberculosis Induces Interleukin-32 Production through a Caspase- 1/IL-18/Interferon-γ-Dependent Mechanism

**DOI:** 10.1371/journal.pmed.0030277

**Published:** 2006-08-15

**Authors:** Mihai G Netea, Tania Azam, Eli C Lewis, Leo A. B Joosten, Maorong Wang, Dennis Langenberg, Xianzhong Meng, Edward D Chan, Do-Young Yoon, Tom Ottenhoff, Soo-Hyun Kim, Charles A Dinarello

**Affiliations:** 1 Division of Infectious Diseases, University of Colorado Health Sciences Center, Denver, Colorado, United States of America; 2 Nijmegen University Center for Infectious Diseases, Nijmegen, Netherlands; 3 Laboratory of Experimental Rheumatology, Radboud University Nijmegen Medical Center, Nijmegen, Netherlands; 4 Division of Surgery, University of Colorado Health Sciences Center, Denver, Colorado, United States of America; 5 Department of Immunohematology and Blood Transfusion, Leiden University, Leiden, Netherlands; 6 Division of Pulmonary Sciences, University of Colorado Health Sciences Center, Denver, Colorado, United States of America; 7 Department of Bioscience and Biotechnology, Konkuk University, Seoul, Korea; 8 Department of Biomedical Science and Technology, Konkuk University, Seoul, Korea; Mahidol University, Thailand

## Abstract

**Background:**

Interleukin (IL)–32 is a newly described proinflammatory cytokine that seems likely to play a role in inflammation and host defense. Little is known about the regulation of IL-32 production by primary cells of the immune system.

**Methods and Findings:**

In the present study, freshly obtained human peripheral blood mononuclear cells were stimulated with different Toll-like receptor (TLR) agonists, and gene expression and synthesis of IL-32 was determined. We demonstrate that the TLR4 agonist lipopolysaccharide induces moderate (4-fold) production of IL-32, whereas agonists of TLR2, TLR3, TLR5, or TLR9, each of which strongly induced tumor necrosis factor α and IL-6, did not stimulate IL-32 production. However, the greatest amount of IL-32 was induced by the mycobacteria Mycobacterium tuberculosis and M. bovis BCG (20-fold over unstimulated cells). IL-32-induced synthesis by either lipopolysaccharide or mycobacteria remains entirely cell-associated in monocytes; moreover, steady-state mRNA levels are present in unstimulated monocytes without translation into IL-32 protein, similar to other cytokines lacking a signal peptide. IL-32 production induced by M. tuberculosis is dependent on endogenous interferon-γ (IFNγ); endogenous IFNγ is, in turn, dependent on M. tuberculosis–induced IL-18 via caspase-1.

**Conclusions:**

In conclusion, IL-32 is a cell-associated proinflammatory cytokine, which is specifically stimulated by mycobacteria through a caspase-1- and IL-18-dependent production of IFNγ.

## Abstract 2

Synthesis of IL-32, a cell-associated proinflammatory cytokine, was promoted by Mycobacterium tuberculosis and M. bovis, suggesting a role in a role in inflammation and host defense against tuberculosis.

## Editors' Summary

**Background.**

Tuberculosis (TB) is a serious infectious disease that is becoming more common. Worldwide it causes around 2 million deaths every year, mostly in developing countries. Some 2 billion people—or one-third of the world's population—are chronically infected without active symptoms. In humans the disease is usually caused by a bacterium called Mycobacterium tuberculosis. Another related bacterium, *Mycobacterium bovis,* causes TB in cattle and sometimes in people.

The immune system, which defends the body against infections, involves a number of cells (for example, the white blood cells) and also chemicals. People with defects in their immune system are more likely to suffer from infectious diseases. Cytokines are one class of chemicals in the immune system. A particular cytokine called interleukin-32 (IL-32) has been shown to play a role in the development of inflammation, which is a part of the body's response to infection. Previous research has suggested that IL-32 might be of particular importance in the defenses against TB. In recent years scientists have discovered a lot about the processes involved in the “switching on” of the individual parts of the immune system in response to infection. However, very little is known about the factors influencing the switching on of production of IL-32.

**Why Was This Study Done?**

It would be useful to know more about the production of IL-32 because it would advance understanding of the immune system in general and, more specifically, how the body protects itself against bacteria, such as those that cause TB.

**What Did the Researchers Do and Find?**

Working with eight healthy volunteers, the researchers took white blood cells of a particular type (peripheral blood mononuclear cells) and exposed them to substances known as TLR agonists. Toll-like receptors (TLRs) are receptors on the surface of leukocytes that recognize specific components of microorganisms. Upon recognition of these microbial components, which function as TLR stimuli (or TLR agonists), signals are transmitted that activate the immune system and thus the host defense. Using a complex series of laboratory procedures, they found that one type of TLR agonist (known as LPS) produced a big increase in IL-32 production, whereas all the other types of TLR agonists that they used produced only small increases. The researchers tested M. tuberculosis and M. bovis bacteria to see whether they increased IL-32 production and they found that they did so, to a greater degree even than LPS. The researchers also learned other details about IL-32 and the pathway of chemical changes that eventually leads to its production.

**What Do These Findings Mean?**

The researchers say their study provides several important insights into the biology of IL-32. The findings confirm that IL-32 is an important factor in the body's defenses against TB. This information will help in understanding how the disease spreads and who is most vulnerable to it. Ultimately, it may assist in the search for new ways of treating and preventing the disease.

**Additional Information.**

Please access these Web sites via the online version of this summary at http://dx.doi.org/10.1371/journal.pmed.0030277.

- The online encyclopedia Wikpedia has useful information on tuberculosis
- Wikipedia also has useful information on the immune system
- More detailed information about international efforts to control TB may be found at the Web sites of the International Union Against Tuberculosis and Lung Disease and the World Health Organization's Stop TB Department

## Introduction

Interleukin (IL)–32 is a recently described cytokine initially thought to be a product of T, natural killer, and epithelial cells. The cytokine exhibits several properties of a classical proinflammatory mediator [1]. For example, IL-32 induces the production of tumor necrosis factor (TNF) α, IL-8, and MIP-2 via the activation of NF-kB and p38 MAP kinase [1]. In addition, maturation of IL-1β through a caspase-1-dependent mechanism is also a property of IL-32 [2]. These proinflammatory effects of IL-32 suggest an important role of IL-32 in inflammation and antibacterial defense.

In macrophages infected with intracellular pathogens, activation by interferon-γ (IFNγ) in combination with TNF is a major effector mechanism of cell-mediated immunity [3]. IFNγ induces antimicrobial killing mechanisms in monocytes, macrophages, and neutrophils, by activating phagocytosis, stimulating oxygen radical production, and activating the synthesis of the inducible form of NO [4,5]. The crucial role of these IFNγ properties for host antimicrobial defense has been demonstrated in patients with defects in either IFNγ or IL-12 receptors [6,7]. These patients have increased susceptibility to intracellular microorganisms such as mycobacteria and salmonella species [6,7]. Infection with atypical mycbobacteria such as non-tuberculous mycobacteria or Mycobacterium bovis BCG is particularly dangerous in patients lacking adequate IFNγ production and is a main factor determining the long-term prognosis of these patients [8].

Because of the proinflammatory effects of IL-32 and its induction from epithelial cell lines by IFNγ [1], we wanted to determine whether IL-32 is produced from primary cells of the immune system in response to stimulation with mycobacteria. We investigated the stimulation of IL-32 synthesis by several agonists of TLR, as well as bacterial pathogens. In the present study, we also define the IFNγ-dependent pathway of M. tuberculosis–induced IL-32 from peripheral blood mononuclear cells (PBMCs).

## Methods

### Reagents and Microorganisms

Synthetic Pam3Cys was purchased from EMC Microcollections (Tubingen, Germany). Lipopolysaccharide (LPS) (Escherichia coli serotype 055:B5) and poly I:C were purchased from Sigma Chemical (St. Louis, Missouri, United States). H37Rv and M. bovis BCG were grown to mid-log phase in Middlebrook 7H9 liquid medium supplemented with oleic acid, albumin, dextrose, and catalase (Difco Laboratories, Sparks, Maryland, United States), washed three times in sterile saline, and resuspended in RPMI 1640 medium. Separate culture suspensions were sonicated for 10 min on ice, in order to obtain noninfective cell lysates. Cultures of Staphylococcus aureus were grown in basic medium broth (Luria broth supplemented with 0.1% K_2_HPO_4_ and 0.1% glucose), washed in sterile saline, and resuspended in RPMI 1640 medium, as described above.

### Stimulation of Whole Blood Cultures with Mycobacteria

After informed consent, venous blood was drawn from the antecubital vein of eight healthy volunteers into heparin tubes. Then 750 μl of either RPMI culture medium or RPMI containing sonicated M. tuberculosis or BGC (both at a concentration of 10 μg/ml) was added to 4.0-ml screw cap cryogenic tubes (Corning, Corning, New York, United States). Thereafter, 250 μl of freshly obtained heparinized blood was added to tubes containing stimuli. The tubes were capped tightly and incubated at 37 °C in an upright position. After 24 h of incubation, the blood was mixed by rapid vortex and transferred into 1.5-ml Eppendorf tubes (Brinkmann Instruments, Westbury, New York, United States). Triton X-100 (Bio-Rad Laboratories, Hercules, California, United States) was then added (0.5% final concentration) and the blood mixed until lysed. The lysed blood was stored at −70 °C until assay. In these whole blood cultures, intracellular and secreted cytokine concentrations were determined together, and the results represent “total” cytokine production.

### Isolation of PBMCs and Stimulation of Cytokine Production

The PBMC fraction was obtained by density centrifugation of blood diluted 1:1 in pyrogen-free saline over Histopaque (Sigma, St. Louis, Missouri, United States). Cells were washed twice in saline and suspended in culture medium (RPMI 1640) supplemented with penicillin (100 U/ml) and streptomycin (100 μg/ml).

To flat-bottom 96-well plates were added 5 × 10^5^ PBMCs in a 100-μl volume, which was then incubated with either 100 μl of culture medium (negative control) or 100 μl of the various stimuli: synthetic Pam3Cys lipopeptide (5 μg/ml), poly I:C (5 μg/ml), E. coli LPS (100 ng/ml), or 1 × 10^7^ heat-killed microorganisms/milliliter of *M. tuberculosis, M. bovis* BCG, or S. aureus. Each Toll-like receptor (TLR) ligand, except LPS, was checked for the presence of LPS in the LAL assay and found to be negative. After 24 h of incubation at 37 °C, supernatants were removed in some experiments. Otherwise, the cells were lysed by 1% Triton X-100 and two cycles of freeze–thaw. Intracellular and secreted cytokine concentrations were determined together, and the results represent “total” cytokine production. In additional experiments, the effect of a caspase-1 inhibitor (20 μM of Ac-Tyr-Val-Ala-Asp-2,6-dimethylbezoyloxymethylketone, YVAD, Alexis Biochemicals, San Diego, California, United States), IL-1 receptor antagonist (IL-1Ra, 10 μg/ml, R&D Systems, Minneapolis, Minnesota, United States), or IL-18 binding protein (10 μg/ml) [9] on the M. tuberculosis–induced production of IL-32 was investigated.

In order to test whether the monocyte or lymphocyte population was the source of IL-32 after stimulation with mycobacteria, PBMCs were incubated for 2 h at 37 °C. The nonadherent cells, transferred into another well, were mostly lymphocytes, whereas the adherent cells were more than 90% monocytes. The stimulation of IL-32 by the mycobacteria was compared between monocytes and lymphocytes.

Production of IL-32 stimulated by mycobacteria was also investigated in monocyte-derived macrophages and dendritic cells (DCs). Adherent monocytes were incubated in RPMI for 5 d in the presence of either 20% heat-inactivated human serum for macrophage differentiation, or a combination of 50 ng/ml GM-CSF (Peprotech, Rocky Hill, New Jersey, United States) plus 20 ng/ml IL-4 (Peprotech) used for DC differentiation. After 5 d, the cells were washed, and the macrophages or DCs were stimulated for an additional 24 h with either M. tuberculosis or M. bovis BCG.

### Cytokine Measurements

TNFα, IL-6, and IFNγ concentrations were determined by specific electrochemiluminescence (ECL) assays, as previously described [10]. A specific ECL assay was developed for the measurement of IL-32. The IgG fraction of polyclonal antibodies produced in goats against recombinant human IL-32α was purified with Econo-Pac (Cooper, Boynton Beach, Florida, United States). This IgG fraction was then affinity purified using IL-32α immobilized on Affi-gel 15 agarose beads (Bio-Rad Laboratories). One aliquot of the affinity-purified anti-IL-32α antibody was labeled with biotin, and another aliquot with ruthenium according with the manufacturer's instructions (BioVeris, Gaithersburg, Maryland, United States). A standard curve was constructed using recombinant human IL-32α (Peprotech). For the assay, 25 μl of a sample or standard was added to 5-ml polypropylene tubes (Becton Dickinson, Franklin Lakes, New Jersey, United States) and incubated with dilutions of the biotinylated and the ruthenylated antibodies for a total aqueous phase of 100 μl and 25 μl of Dynabeads (BioVeris). After shaking at room temperature for 4 h, the reaction was terminated with 200 μl of phosphate-buffered saline (PBS). The electrochemiluminescent signal was measured using an Origen Analyzer (BioVeris), and the concentrations of IL-32 were calculated from the standard curve generated for each assay. In over ten assays, the ECL assay for IL-32 detected from 3 pg/ml to 2,000 pg/ml. Because of the polyclonal nature of the goat antibodies and the overlapping amino acids of the four IL-32 isoforms, all isoforms of IL-32 were likely detected in the ECL assay.

### RT-PCR for the IL-32 Isoforms

Ten million freshly isolated PBMCs were stimulated with either culture medium, 100 ng/ml LPS, or 1 × 10^7^ heat-killed M. tuberculosis. After 4 h incubation at 37 °C, total RNA was extracted in 1 ml of TRIzol reagent (Invitrogen, Carlsbad, California, United States), an improved single-step RNA isolation method based on the method described by Chomczynski and Sacchi [11]. In addition, RNA was also isolated from an aliquot of freshly obtained PBMCs, without any culture or stimulation. Thereafter, RNA was precipitated with isopropanol, washed with 70% ethanol, and dissolved in water. Isolated RNA was treated with DNase before being reverse transcribed into complementary DNA using oligo(dT) primers and MMLV reverse transcriptase. PCR was performed using a Peltier Thermal Cycler-200 (Watertown, Massachusetts, United States). Primer sequences for the IL-32 isoforms are presented in Table 1: one set of primers detected both isoform α and β, whereas two different sets of primers detected isoforms γ and δ. GAPDH was used as a reference gene, for which the primers were 5′-GGC AAA TTC AAC GGC ACA-′3 (forward) and 5′-GTT AGT GGG GTC TCG CTC TG-′3 (reverse). PCR conditions were as follows: 2 min at 50 °C and 10 min at 95 °C, followed by 30 cycles of PCR reaction at 94 °C for 45 s, 70 °C for 2 min, and 59 °C for 1 min. The PCR products were run on 1% agarose gels stained with ethidium bromide.

**Table 1:**
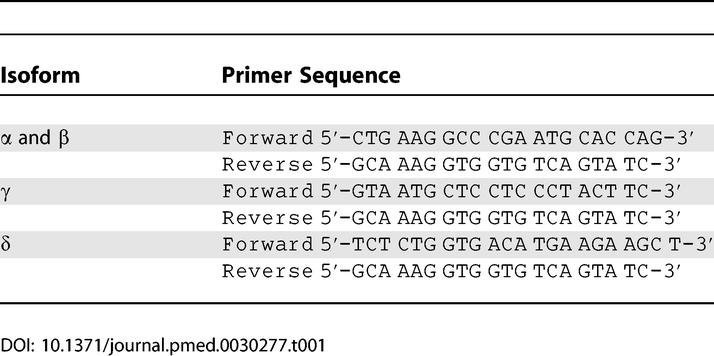
Primer Sequences Used in the RT-PCR for the Four Isoforms of IL-32

### Immunostaining

Two distinct methods were used for immunostaining, confocal microscopy, and optical microscopy. For confocal microscopy, human PBMCs were isolated and cultured on eight-chamber glass slides (Nalge Nunc International, Naperville, Illinois, United States) at 37 °C in a humidified atmosphere of 5% CO_2_. After the cells were exposed to appropriate incubation conditions, they were fixed in 70% acetone and 30% methanol for 5 min and washed in PBS. Then the cells were fixed in 4% paraformaldehyde for 10 min at room temperature and washed with PBS. The fixed cells were permeabilized in PBS containing 5% donkey normal serum for 30 min at room temperature. This was followed by exposure to the primary mouse anti-human IL-32 monoclonal IgG_1_ antibody at 4 °C overnight. As negative control, mouse IgG_1_ was used instead of the anti-IL-32 antibody. After being washed in PBS, the cells were counterstained with the secondary donkey anti-mouse antibody linked to fluorescein-labeled Cy3 (1:150, red), wheat germ agglutinin (5 μg/ml, green for cell surface staining), and AMCA(33342) (50 μl/ml, blue) for 45 min at room temperature. Then the slides were mounted with aqueous antiquenching medium. The PBMCs were examined by digital deconvoluted microscopy.

For optical microscopy, PBMCs were isolated and stimulated for 24 h in the presence of culture medium, LPS (100 ng/ml), or heat-killed M. tuberculosis (10 μg/ml) using TEFLON-PFA six-well format culture disks (Savillex, Minnetonka, Minnesota, United States) to avoid adherence of the PBMCs. After 24 h, cytospins of the stimulated cells were prepared and stained with the monoclonal anti-IL-32 antibody (MoAb, clone 9). The primary antibody (5 mg/ml) was diluted (1:500) and incubated for 30 min with the tissue sections, followed by rabbit anti-mouse HRPO (1:200, Dako, Glostrup, Denmark) for 60 min. The slides were then sequentially incubated with the chromogen (DAB) reagent for 10 min, counterstained with Mayer's hematoxylin, and mounted in permount. Negative control staining was performed using mouse IgG_1_ isotype antibody. A Leica (Wetzlar, Germany) microscope (DMRD model) equipped with a digital camera was used to prepare the microphotographs**.**

### Statistical Analysis

The human experiments were performed in duplicate with blood obtained from eight volunteers. The differences between groups were analyzed by Mann–Whitney *U* test. The data are given as means ± standard error of the mean (SEM).

## Results

### Stimulation of IL-32 by Mycobactyeria

**Figure 1:**
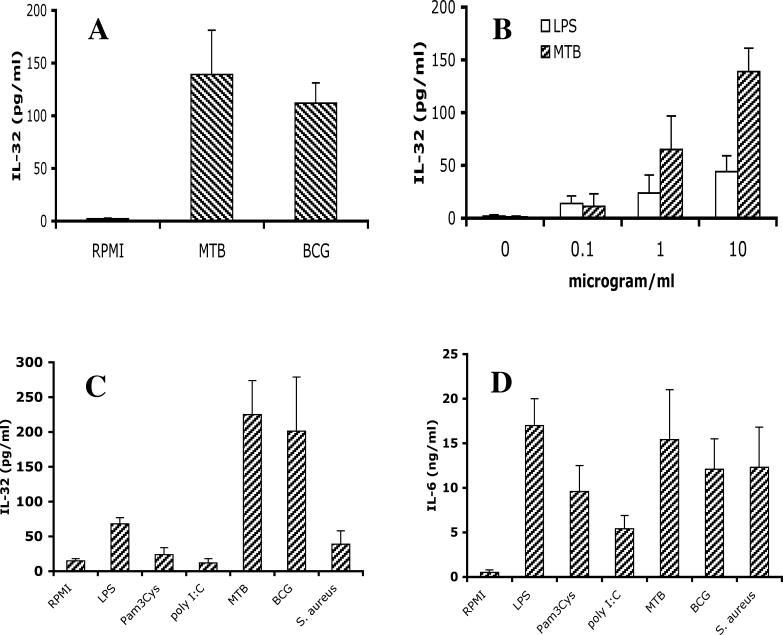
Stimulation of IL-32 by Mycobacteria (A) Whole blood was diluted 1:4 with RPMI and stimulated with 10 μg/ml sonicated M. tuberculosis (MTB) or M. bovis BCG (BCG). Triton X-100 (0.5%) was added 24 h later, and IL-32 concentration was measured by ECL. (B) Stimulation of freshly isolated PBMCs with various concentrations of LPS or sonicated M. tuberculosis stimulated IL-32 synthesis in a dose-dependent manner. (C and D) Freshly isolated PBMCs were stimulated with TLR4 (LPS, 100 ng/ml), TLR2 (Pam3Cys, 10 μg/ml), TLR3 (poly I:C, 5 μg/ml), M. tuberculosis, M. bovis BCG, or S. aureus (all at 1 × 10^7^ microorganisms/milliliter, heat-killed). After 24 h of stimulation, LPS-, *M. tuberculosis–,* and M. bovis BCG–stimulated production of IL-32 (C) or IL-6 (D) was measured by ECL. Data are presented as means ± SEM (*n =* 8).

Whole blood diluted one in four with RPMI was stimulated with 10 μg/ml heat-killed M. tuberculosis or M. bovis BCG. After 24 h, there was significant stimulation of IL-32 as measured in the total cell lysate of these cultures (Figure 1A). The level of IL-32 in the lysates of whole blood cultures without stimulation was less than 5 pg/ml, whereas M. tuberculosis and M. bovis BCG increased total IL-32 synthesis over 30-fold.

As shown in Figure 1B, a dose-dependent stimulation of IL-32 release was observed when freshly isolated PBMCs were stimulated with M. tuberculosis. Interestingly, LPS also stimulated moderate amounts of IL-32 in PBMCs in a dose-dependent fashion, but these amounts were clearly lower than those induced by M. tuberculosis (Figure 1B)*.* In contrast, stimulation with other TLR agonists such as Pam3Cys (TLR2) and poly I:C (TLR3) did not induce IL-32, despite incremental stimulations with concentrations of the stimuli up to 100 μg/ml (Figure 1C and data not shown). The TLR agonists flagellin (TLR5) and CpG (TLR9) did not induce IL-32 either (data not shown). Similarly, stimulation of PBMCs with other heat-killed microorganisms such as S. aureus (shown in Figure 1C), *Candida albicans,* or Aspergillus fumigatus (data not shown) did not stimulate IL-32 production. In contrast, all the TLR agonists and microorganisms described above were potent inducers of IL-6 (Figure 1D) and TNF (not shown) production, in ranges similar to those induced by M. tuberculosis or LPS.

Unlike PBMC cultures, whole blood cultures contain all the plasma components including antibodies and macromolecules such as complement components and anti-proteases. Whereas PBMCs are composed of 10%–15% monocytes and mostly lymphocytes, whole blood cultures contain 60%–70% neutrophils, few monocytes (4%–6%), and about 20% lymphocytes. When comparing total IL-32 production stimulated with either M. tuberculosis or M. bovis BCG, whole blood cultures produced 136 pg per million monocytes, whereas in PBMC cultures, 420 pg were produced per million monocytes.

### IL-32 Induced by Inflammatory Stimuli Is a Cell-Associated Cytokine

**Figure 2:**
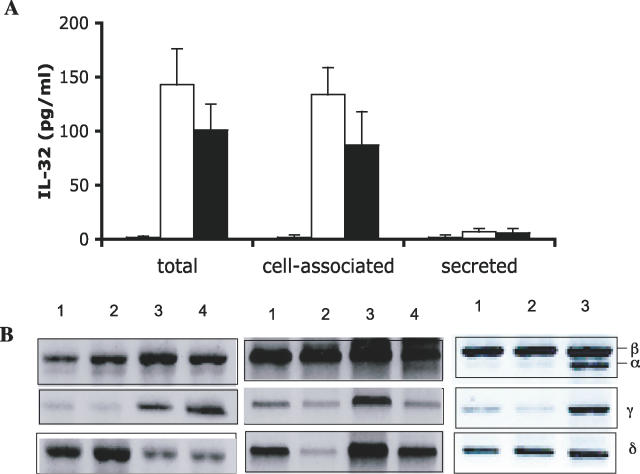
IL-32 Induced by Inflammatory Stimuli Is a Cell-Associated Cytokine (A) Human PBMCs were stimulated with 10 μg/ml sonicated M. tuberculosis. After 24 h, supernatants and cell lysates were assayed for IL-32 (see Methods). (B) RT-PCR of the four IL-32 isoforms in freshly lysed PBMCs (lane1), PBMCs incubated in medium for 4 h (lane 2), stimulated with 100 ng/ml LPS (lane 3), or with M. tuberculosis (lane 4).

When human PBMCs were stimulated with 1 × 10^7^ microorganisms/milliliter of heat-killed *M. tuberculosis,* nearly all IL-32 measured was found in the cell-associated fraction and not the supernatant medium (Figure 2A). IL-32 was not detectable in freshly lysed PBMCs or in PBMCs incubated for 24 h without added stimulants. However, as shown in Figure 2B, steady-state levels of mRNA for IL-32β, IL-32γ, and IL-32δ are present in freshly isolated PBMCs without adherence to culture vessels. Levels of IL-32α are low but present. After 4 h of incubation with RPMI only, the levels of mRNA coding for each of the isoforms remained unchanged (Figure 2B). Upon stimulation with LPS for 24 h, there were markedly increased levels of IL-32α and IL-32γ, but little change in IL-32β and IL-32δ (Figure 2B).

**Figure 3:**
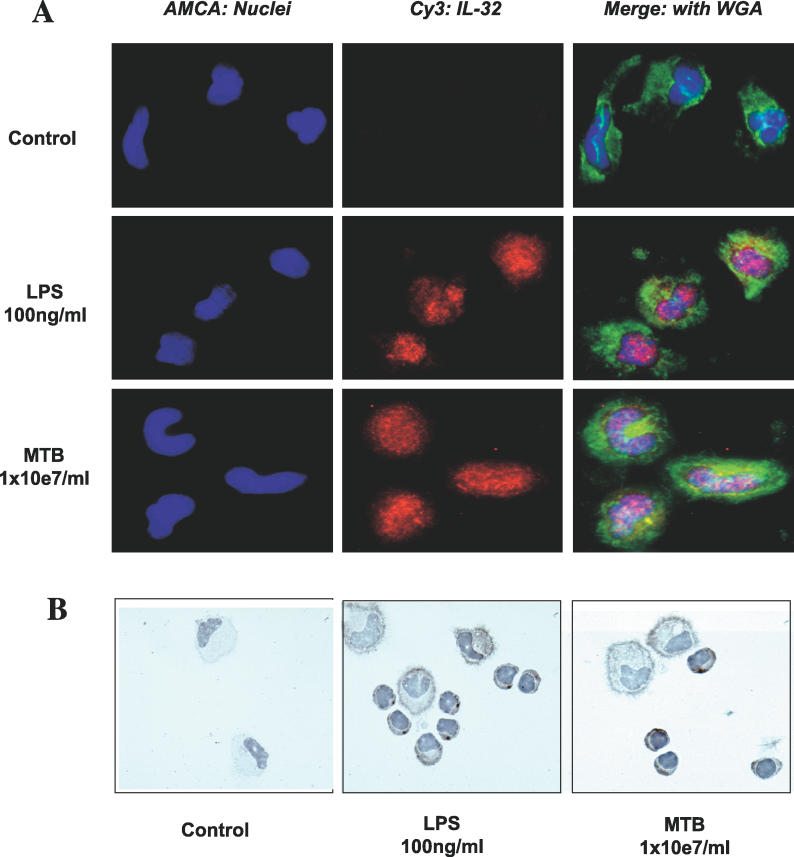
IL-32 Immunostaining of Human PBMCs Stimulated with LPS or M. tuberculosis (A) Freshly isolated PBMCs were left to adhere, stimulated with either control medium (first row), LPS (second row), or M. tuberculosis (MTB; third row), and 24 h later stained for the presence of IL-32. Nuclei were stained with AMCA (blue, left panels), and IL-32 was stained with Cy3 (red, middle panels). The right panels show the merged pictures with WGA-stained cellular surface (green).The stained cells were examined by digital deconvoluted microscopy. (B) PBMCs stimulated with control medium (left panel), LPS (middle panel), or *M. tuberculosis* (right panel) were stained with an anti-IL-32 monoclonal antibody and counterstained with Mayer's hematoxylin for optical microscopy.

As shown in Figure 3A, immunostaining with a monoclonal mouse anti-human IL-32 antibody also shows the absence of IL-32 protein in PBMC cultures after 24 h without added stimulants (Figure 3A, upper lane, and Figure 3B, left panel). However, IL-32 was present after 24 h of incubation with LPS (Figure 3A, middle lane) or M. tuberculosis (Figure 3A, lower lane). In addition, immunohistochemistry of the cytospins demonstrates that stimulation by LPS or M. tuberculosis revealed a pattern of IL-32 staining particularly associated with the cell membrane (Figure 3B, middle and right panels).

### Monocytes Are the Source of IL-32, but the Presence of Lymphocytes Increases IL-32 Production

**Figure 4:**
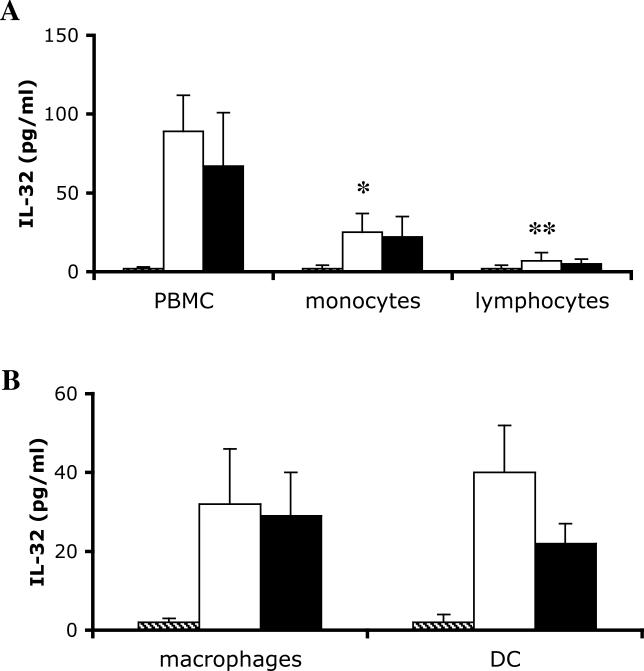
IL-32 Production by Monocytes, Macrophages, and DCs (A) PBMCs were incubated for 2 h at 37 °C in 96-well plates, and non-adherent lymphocytes were transferred to a different well. The total PBMCs, the adherent monocytes, or the non-adherent lymphocytes were stimulated with RPMI (hatched bars), M. tuberculosis (open bars), or M. bovis BCG (solid bars) for an additional 24 h. After 24 h, IL-32 concentrations were measured. (B) Adherent monocytes were incubated for 5 d with RPMI and 20% human serum for macrophage differentiation, or a combination of 50 ng/ml GM-CSF and 20 ng/ml IL-4 for DC differentiation. The macrophages and DCs were thereafter stimulated for an additional 24 h with RPMI (hatched bars), M. tuberculosis (open bars), or M. bovis BCG (solid bars). IL-32 concentration was measured by ECL. Data are presented as means ± SEM (*n* = 8); *, *p* < 0.05; **, *p* < 0.01.

PBMCs were separated into adherent monocytes, and non-adherent lymphocytes, which were removed and cultured separately. When stimulated with M. tuberculosis or M. bovis BCG, monocytes were the primary source of IL-32, as demonstrated by the significantly higher concentration of IL-32 in the cell lysates of stimulated monocytes, compared with the lymphocytes (Figure 4A). However, comparison to the mixture of monocytes and lymphocytes in unfractionated PBMCs makes it clear that the presence of lymphocytes potentiates the stimulation of IL-32 by the monocytes, and thus contributes significantly to the total production of IL-32 (Figure 4A). Each volunteer tested in the present study was PPD-negative, which rules out the influence of lymphocytes due to antigen presentation of mycobacteria to specific T cells.

To investigate the production of IL-32 by differentiated monocytes, freshly obtained blood monocytes were cultured with 20% human serum to induce macrophage-like cells, or with a combination of GM-CSF/IL-4 to obtain a DC population. As shown in Figure 4B, monocyte-derived macrophages and DCs produced approximately the same amount of IL-32 as freshly cultured adherent monocytes (see Figure 4A). This was observed for both M. tuberculosis and M. bovis BCG stimulation.

### IFNγ Induces Production of IL-32 in Primary Cells

**Figure 5:**
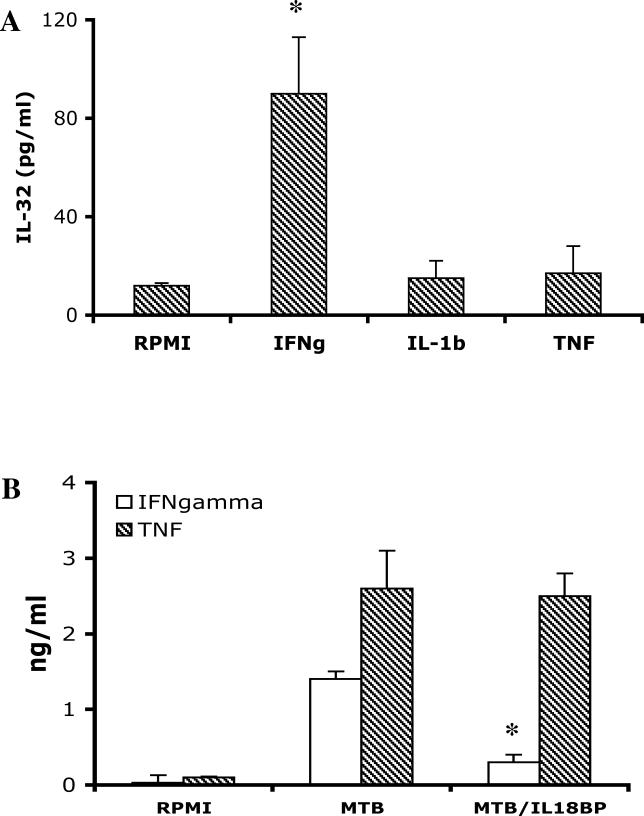
IFNγ Is a Stimulus of IL-32 Production (A) PBMCs isolated from five volunteers were stimulated with IFNγ, IL-1β, or TNFα (all at a concentration of 10 ng/ml). IL-32 concentrations were measured by ECL 24 h later in cells lysed with Triton X-100. (B) PBMCs were stimulated for 24 h with RPMI, M. tuberculosis (MTB), or M. tuberculosis in combination with 10 μg/ml IL-18BP. IFNγ and TNFα concentrations were measured 24 h later. Data are presented as means ± SEM (*n =* 5); *, *p* < 0.05.

As shown in Figure 5, PBMCs stimulated with IFNγ induced an 8-fold increase in IL-32. In contrast, IL-1β or TNFα did not stimulate the production of IL-32 in PBMCs, although these cytokines (similar to IFNγ) are able to induce IL-32 in epithelial cells [1]. As a control, in the same PBMC cultures IL-1β and TNFα induced chemokines and IL-6, whereas IFNγ did not (data not shown). Therefore, it appears that IFNγ is a highly selective IL-32-inducing cytokine for blood monocytes.

We have compared the production of IFNγ induced by the various stimuli used for the induction of IL-32 release. The highest amounts of IFNγ production were stimulated by M. tuberculosis (987 ± 185 pg/ml) and M. bovis BCG (794 ± 201 pg/ml), whereas LPS (217 ± 44 pg/ml), S. aureus (343 ± 101 pg/ml), and C. albicans (197 ± 88 pg/ml) induced moderate amounts of the cytokine. No IFNγ production was induced by Pam3Cys or poly I:C (data not shown). These data suggest that the induction of IL-32 by mycobacteria and LPS may be at least in part mediated by endogenous IFNγ release.

In human PBMCs stimulated with heat-killed S. epidermidis or LPS, IFNγ production is dependent on IL-18, since in the presence of IL-18BP IFNγ production is reduced by over 80% [12]. The role of IL-18 in IFNγ production by microbial products is dependent on processing and release of IL-18 by caspase-1 [13,14]. As shown in Figure 5B, M. tuberculosis–induced IFNγ production was reduced 80% by the neutralization of endogenous IL-18 by IL-18BP. In contrast, the production of TNFα was unaffected by the neutralization of endogenous IL-18 (Figure 5B).

### M. tuberculosis Stimulates IL-32 through a Caspase-1/IL-18/IFNγ-Dependent Mechanism

**Figure 6:**
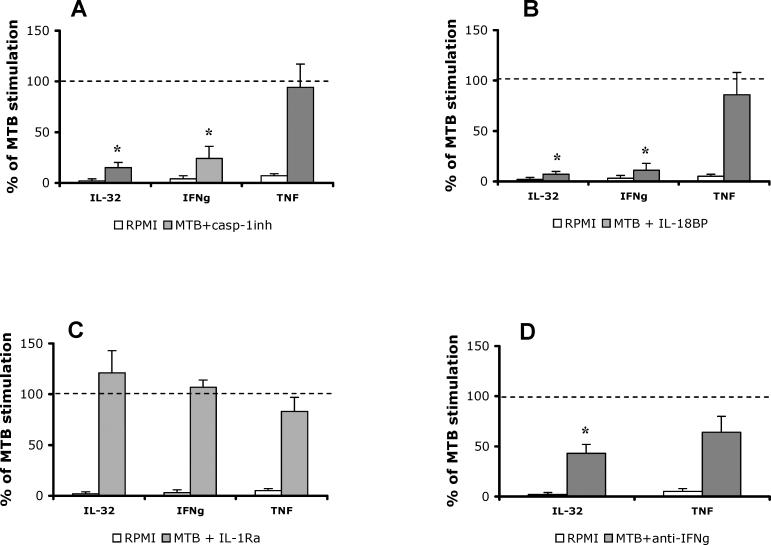
M. tuberculosis Stimulates IL-32 through a Caspase-1/IL-18/IFNγ-Dependent Mechanism PBMCs were stimulated with RPMI, M. tuberculosis (MTB), or a combination of M. tuberculosis and cytokine inhibitors: 20 μM of a caspase-1 inhibitor (casp-1inh) (A), 10 μg/ml IL-18BP (B), IL-1Ra (C), or 10 μg/ml of a monoclonal mouse anti-human IFNγ antibody (D). As a control for the anti-IFNγ antibody, a similar concentration of a purified mouse IgG1 antibody was used in the control stimulation. After 24 h of incubation, IL-32, IFNγ, and TNF concentrations were measured by ECL. The dashed line represents the 100% stimulation induced by M. tuberculosis alone. The open bars represent the unstimulated cells, and the solid bars represent the cells stimulated with M. tuberculosis in the presence of the cytokine inhibitors, expressed as percent of M. tuberculosis production. Data are presented as means ± SEM (*n =* 8); *, *p* < 0.05.

As shown in Figure 6A, the production of IL-32 in PBMCs stimulated with M. tuberculosis was reduced by 83% in the presence of the caspase-1 inhibitor. Under these same conditions IFNγ levels were reduced by 77%, but TNFα production was unaffected by the inhibition of caspase-1. To determine whether inhibition of caspase-1 was reducing the activity of endogenous IL-18 or IL-1β, the PBMCs were stimulated with M. tuberculosis in the presence of their respective natural inhibitors, IL-18BP and IL-1Ra. As shown in Figure 6C, blocking IL-1 activity with saturating concentrations of IL-1Ra had no effect on the production of IL-32, IFNγ, or TNFα, whereas IL-6 production was reduced by 44% (*p* < 0.05; data not shown). As expected, in these same cultures, IL-18BP reduced both IL-32 (92% reduction) and IFNγ (88% reduction), but not TNFα (Figure 6B). Although we observed that exogenous IFNγ induced IL-32 in PBMCs (Figure 5A), in order to assess whether endogenous IFNγ provided a similar signal, IFNγ activity was neutralized with neutralizing anti-IFNγ antibody. Blockade of endogenous IFNγ activity induced by M. tuberculosis strongly inhibited the production of IL-32 (56% reduction; Figure 6D). Not unexpectedly, reducing IFNγ activity also moderately reduced TNFα synthesis, although this reduction did not reach statistical significance (Figure 6D). Collectively, these experiments demonstrate that M. tuberculosis stimulates the production of IL-32 through a caspase-1/IL-18/IFNγ pathway.

## Discussion

In the present study we demonstrate that mycobacteria species such as M. tuberculosis or M. bovis BCG are potent stimuli for the production of the newly described proinflammatory cytokine IL-32. Steady-state levels of mRNA for IL-32 were present in freshly obtained blood PBMCs in the absence of culture or stimulants in healthy individuals, but also after 24 h of incubation. LPS and M. tuberculosis increased steady-state levels of IL-32 mRNA for isoforms α and γ of the cytokine. Although the ability of LPS to induce the synthesis of IL-32 was moderate (3- to 4-fold increase), the mycobacteria M. tuberculosis and M. bovis BCG induced up to a 20-fold increase in IL-32 synthesis over PBMCs cultured in the absence of mycobacteria. Furthermore, we demonstrated that the increase in IL-32 production by mycobacteria is dependent of the proinflammatory pathway involving active IL-18 induced by a caspase-1-dependent pathway. However, the role for IL-18 is primarily the production of IFNγ, which in turn stimulates IL-32. Indeed, neutralization of endogenous IFNγ prevents M. tuberculosis–induced IL-32, and culturing PBMCs with exogenous IFNγ increases IL-32 production 10-fold. We conclude that IFNγ is the ultimate mediator of IL-32 synthesis induced by mycobacteria.

When PBMCs were stimulated with a large variety of TLR agonists and heat-killed microorganisms, mycobacteria were the most potent in inducing the synthesis of IL-32 in both whole blood and freshly isolated PBMCs (Figure 1). Of the TLR agonists, only the TLR4 stimulus LPS was able to stimulate moderate production of IL-32 (3- to 4-fold increase). These data suggest that while LPS/TLR4 interaction can increase transcription of IL-32 mRNA over that present in unstimulated cells, a second signal for synthesis is provided specifically by mycobacteria for the production of the protein. The specificity of IL-32 stimulation by mycobacteria, but not other microorganisms, is clinically significant since IL-32 is an IFNγ-inducible gene, and IFNγ pathway defects are accompanied by an increased susceptibility to infection with low-virulent mycobacteria [6–8]. This specificity suggests a potential role of IFNγ-induced IL-32 in the host defense against mycobacterial infections. The findings are also suggestive of a role of IL-32 in mediating some of the consequences of mycobacterial stimulation. For example, we have reported that IL-32 synergizes specifically with the muramyldipeptide for IL-6 production [2]. The muramyldipeptide is the minimal structure able to reproduce the adjuvant properties of the mycobacterial cell wall, and IL-6 plays a central role in this adjuvant activity [15].

IFNγ induces IL-32 in epithelial cells [1], and we have observed a similar induction in PBMCs. Although other proinflammatory cytokines such as TNF and IL-1β induce IL-32 in epithelial cells [1], this was not observed in PBMCs (Figure 4). We investigated whether stimulation of IL-32 by M. tuberculosis is indeed mediated through synthesis of endogenous IFNγ. Although the monocyte is the primary source of IL-32, lymphocytes are also required for optimal IL-32 synthesis. It is well established that lymphocytes and natural killer cells are the two main cell populations involved in the production and release of IFNγ [16]. Stimulation of IFNγ by M. tuberculosis was dependent on intermediary production of endogenous IL-18 (Figure 4), similarly to the stimulation by other microorganisms [17]. Inhibition of IL-18 processing by caspase-1, or neutralization of IL-18 by IL-18BP, inhibited not only IFNγ production, but also the induction of IL-32 by M. tuberculosis. Because caspase-1 also inhibits processing of pro-IL-1β into an active mature form, it was necessary to exclude a role for endogenous IL-1β in the effects of the caspase-1 inhibitor. Unlike for IL-18, blockade of IL-1 by IL-1Ra had no effect on *M. tuberculosis–*induced IL-32 production. The fact that IL-32 stimulation by M. tuberculosis was mediated by the release of endogenous IFNγ was further demonstrated by the blockade of IL-32 synthesis by a neutralizing antibody to IFNγ. Thus, the chain of events leading to stimulation of IL-32 by M. tuberculosis involves caspase-1-dependent IL-18 from monocytes, which stimulates production of IFNγ from lymphocytes. IFNγ, in turn, drives the translational machinery for IL-32 synthesis in the monocytes and macrophages.

Steady-state levels of mRNA coding for IL-32β, IL-32γ, and IL-32δ are clearly present in freshly obtained PBMCs, whereas gene expression for IL-32α is barely detectable; importantly, there is no detectable IL-32 protein in the lysates of these cells. IL-32 mRNA of isoforms α and γ was stimulated by both LPS and *M. tuberculosis,* while isoform β was quite strongly expressed in steady-state unstimulated cells, and was not further induced by stimulation. In contrast, IL-32 mRNA isoform δ expression was heterogenous, both in terms of basal steady-state expression and response to stimulation. Even after 24 h of culture on a plastic surface, there was no IL-32 protein in the lysates. However, upon stimulation with LPS and mycobacteria, IL-32 protein concentration rose 4-fold and 20-fold, respectively. Since the ECL method for detecting IL-32 does not distinguish between the different isoforms, the IL-32 protein in the lysates could be due to any one of the isoforms.

The dissociated expression of the IL-32 gene and synthesis of the cytokine itself is reminiscent of the dissociation of IL-1β gene expression and synthesis in PBMCs. In studies of IL-1β expression, adherence to glass or plastic in the strict absence of endotoxins or other microbial agents was sufficient for IL-1β gene expression, but not for the translation into the IL-1β precursor [18]. Even using the inflammatory C5a complement component, human PBMCs exhibit the same levels of IL-1β and TNFα gene expression as those induced with LPS [19] but, in the case of C5a stimulation, without detectable translation of IL-1β or TNFα [19]. In those studies, there was no failure of mRNA coding of IL-1β to associate with large polysomes and initiate translation [20]; in fact, evidence revealed that initiation of translation was intact [20]. However, adherence to glass and plastic surfaces or stimulation by C5a was not sufficient to complete the translation into the IL-1β precursor. The failure to complete translation appeared to be due to early termination of elongation of the polypeptide [20]. LPS or IL-1β itself at exceedingly low concentrations provide the intracellular components to complete the translation [19]. It is presently unknown whether mRNA coding for IL-32α, IL-32β, IL-32γ, or IL-32δ is similarly incapable of completing translation or whether binding to the ribosome is defective. However, the data in the present study suggest that unlike most mRNA coding for cytokines, IL-32 mRNA belongs to the class of proinflammatory cytokines that are regulated unless a second signal is provided. In addition, stronger IFNγ stimulation by mycobacteria compared to other stimuli could also have played a role in the differential stimulation of IL-32. However, IFNγ and IL-32 production were not completely correlated, as some microorganisms such as S. aureus or C. albicans induced IFNγ production, yet they were incapable of stimulating IL-32 release. In this respect, the release of intermediary IFNγ seems to be a necessary, but not sufficient, step in the induction of IL-32.

Corroborating these findings is the absence of IL-32 protein in adherent blood monocytes cultured for 24 h and examined by immunostaining with an anti-human IL-32 monoclonal antibody. The absence of IL-32 protein employing either an affinity-purified goat anti-human IL-32α antibody or the monoclonal antibody supports the concept that IL-32 gene expression can exist without translation into IL-32 protein. The monoclonal antibody's ability to detect IL-32 was apparent following stimulation with LPS or M. tuberculosis. There was staining for IL-32 on the cells' surface and measurable IL-32 in the lysates of these cells. The staining of IL-32 in the confocal microscopy was comparable to the diffuse cytosolic staining patterns seen for IL-1α and IL-1β in LPS-stimulated monocytes [21]. However, the localized immunohistochemical staining of the cell membrane after stimulation with LPS or M. tuberculosis suggests that IL-32 is a cell-associated cytokine, similar to other cytokines such as IL-1α or lymphotoxin-α [22,23].

Both IL-32 (all isoforms) and IL-1β lack a classic signal peptide. Therefore, it is not unexpected that in stimulated blood monocytes there is no detectable IL-32 in the supernatant whereas the cell lysates contain nearly all of the measurable IL-32. Because LPS and M. tuberculosis stimulate caspase-1 cleavage of IL-1β, the lack of secreted IL-32 is consistent with the absence of a caspase-1 site in any of the isoforms of IL-32. In fact, the decrease in IL-32 synthesis in the presence of a caspase-1 inhibitor is not a direct effect of caspase-1 on IL-32 but rather on IL-18-induced IFNγ production. In addition, there is no structural relationship between members of the IL-1 family and IL-32, also supporting the absence of caspase-1 cleavage sites in IL-32.

Several cytokines and growth factors lack a clear signal peptide and are not secreted, but nevertheless play a role in the pathogenesis of disease. For example, IL-1α is not secreted from monocytes following stimulation by LPS and other TLR agonists, despite the presence of large intracellular stores. Yet, IL-1α plays an important role in immune responses to specific antigens. Comparing mice deficient in IL-1α or IL-1β, IL-1α but not IL-1β was required for contact-allergen-specific T cell activation during the sensitization phase in contact hypersensitivity [24]. In addition, macrophages of mice deficient in IL-1α display defective phagocytosis and killing of *C. albicans,* which contributes to their increased susceptibility to disseminated infections in these mice (A. G. Vonk, M. G. Netea, J. H. van Krieken, Y. Iwakura, J. W. van der Meer, et al., unpublished data). Similarly, membrane-bound TNFα plays an important role in the pathogenesis of tuberculosis [25]. Another cytokine, acidic fibroblast growth factor also lacks a signal peptide and is found primarily in the cytosol. Yet, acidic fibroblast growth factor plays an important role in neural cell growth [26]. Similarly, although IL-32 appears to be mainly a cell-associated cytokine, its proinflammatory properties suggest important effects on inflammation and host defense.

An important issue relevant to the present study is the identity of the pattern-recognition receptors that trigger the translation the of IL-32 mRNA following stimulation with mycobacteria. The induction of IL-32 by LPS suggests that TLR4 may be involved, and this is consistent with the studies showing a role of TLR4 in the recognition of mycobacteria [27]. However, TLR4 alone is unlikely to be the sole pathway needed for IL-32 stimulation, given the less efficient induction of IL-32 by LPS compared with M. tuberculosis and M. bovis BCG. It is therefore likely that a secondary pathway is activated by mycobacteria, which is needed in combination with TLR4 stimulation for the induction of IL-32. Further studies are needed to elucidate this aspect.

In conclusion, the results of the present study give several important insights into the biology of IL-32. Firstly, mycobacteria species induce production of IL-32 from human monocytes and macrophages, while other microorganisms such as S. aureus and the fungal pathogens *C. albicans* and A. fumigatus do not. Secondly, this capacity of mycobacteria to stimulate IL-32 is mediated by their capacity to induce IFNγ, a crucial cytokine of host defense against these microorganisms. Furthermore, IL-32 is a cell-associated cytokine, and in this form may exert its proinflammatory effects.

## Abbreviations

DC: dendritic cell
ECL: electrochemiluminescence
IL: interleukin
INFγ: interferon-γ
LPS: lipopolysaccharide
PBMC: peripheral blood mononuclear cell
PBS: phosphate-buffered saline
SEM: standard error of the mean
TLR: Toll-like receptor
TNF: tumor necrosis factor

## References

[pmed-0030277-b001] Kim SH, Han SY, Azam T, Yoon DY, Dinarello CA (2005). Interleukin-32: A cytokine and inducer of TNF-alpha. Immunity.

[pmed-0030277-b002] Netea MG, Azam T, Ferwerda G, Girardin SE, Walsh M (2005). IL-32 synergizes with nucleotide oligomerization domain (NOD) 1 and NOD2 ligands for IL-1beta and IL-6 production through a caspase 1-dependent mechanism. Proc Natl Acad Sci U S A.

[pmed-0030277-b003] Ottenhoff TH, Verreck FAW, Hoeve MA, van de Vosse E (2005). Control of human host immunity to mycobacteria. Tuberculosis.

[pmed-0030277-b004] Murray HW, Scavuzzo D, Jacobs JL, Kaplan MH, Libby DM (1987). In vitro and in vivo activation of human mononuclear phagocytes by interferon-g. J Immunol.

[pmed-0030277-b005] Perrusia B, Kobayashi M, Rossi ME, Anegon I, Trinchieri G (1987). Immune interferon enhances functional properties of human granulocytes: Role of Fc receptors and effect of lymphotoxin, tumor necrosis factor, and granulocyte-macrophage colony-stimulating factor. J Immunol.

[pmed-0030277-b006] Ottenhoff TH, Verreck FA, Lichtenauer-Kaligis EG, Hoeve MA, Sanal O (2002). Genetics, cytokines and human infectious disease: Lessons from weakly pathogenic mycobacteria and salmonellae. Nat Genet.

[pmed-0030277-b007] Doffinger R, Dupuis S, Picard C, Fieschi C, Feinberg J (2001). Inherited disorders of IL-12- and IFNg-mediated immunity: A molecular genetics update. Mol Immunol.

[pmed-0030277-b008] van de Vosse E, Hoeve MA, Ottenhoff TH (2004). Human genetics of intracellular infectious diseases: Molecular and cellular immunity against mycobacteria and salmonellae. Lancet Infect Dis.

[pmed-0030277-b009] Kim SH, Eisenstein M, Reznikov L, Fantuzzi G, Novick D (2000). Structural requirements of six naturally occurring isoforms of the IL-18 binding protein to inhibit IL-18. Proc Natl Acad Sci U S A.

[pmed-0030277-b010] Puren AJ, Razeghi P, Fantuzzi G, Dinarello CA (1998). Interleukin-18 enhances lipopolysaccharide-induced interferon-gamma production in human whole blood cultures. J Infect Dis.

[pmed-0030277-b011] Chomczynski P, Sacchi N (1987). Single step method of RNA isolation by acid guanidinum phenol chloroform extraction. Anal Biochem.

[pmed-0030277-b012] Stuyt RJ, Netea MG, Kim SH, Novick D, Rubinstein M (2001). Differential roles of interleukin-18 (IL-18) and IL-12 for induction of gamma interferon by staphylococcal cell wall components and superantigens. Infect Immun.

[pmed-0030277-b013] Gu Y, Kuida K, Tsutsui H, Ku G, Hsiao K (1997). Activation of interferon-g inducing factor mediated by interleukin-1beta converting enzyme. Science.

[pmed-0030277-b014] Ghayur T, Banerjee S, Hugunin M, Butler D, Herzog L (1997). Caspase-1 processes IFN-g-inducing factor and regulates LPS-induced IFN-g production. Nature.

[pmed-0030277-b015] Chedid L, Audibert F, Jolivet M (1986). Role of muramyl peptides for the enhancement of synthetic vaccines. Dev Biol Stand.

[pmed-0030277-b016] Okamura H, Kashiwamura S, Tsutsui H, Yoshimoto T, Nakanishi K (1998). Regulation of interferon-gamma production by IL-12 and IL-18. Curr Opin Immunol.

[pmed-0030277-b017] Dinarello CA (1999). IL-18: A Th1-inducing, proinflammatory cytokine and a new member of the IL-1 family. J Allergy Clin Immunol.

[pmed-0030277-b018] Schindler R, Clark BD, Dinarello CA (1990). Dissociation between interleukin-1b mRNA and protein synthesis in human peripheral blood mononuclear cells. J Biol Chem.

[pmed-0030277-b019] Schindler R, Gelfand JA, Dinarello CA (1990). Recombinant C5a stimulates transcription rather than translation of interleukin-1 (IL-1) and tumor necrosis factor: Translational signal provided by lipopolysaccharide or IL-1 itself. Blood.

[pmed-0030277-b020] Kaspar RL, Gehrke L (1994). Peripheral blood mononuclear cells stimulated with C5a or lipopolysaccharide to synthesize equivalent levels of IL-1b mRNA show unequal IL-1b protein accumulation but similar polyribosome profiles. J Immunol.

[pmed-0030277-b021] Andersson J, Björk L, Dinarello CA, Towbin H, Andersson U (1992). Lipopolysaccharide induces human interleukin-1 receptor antagonist and interleukin-1 production in the same cell. Eur J Immunol.

[pmed-0030277-b022] Dinarello CA (1996). Biologic basis for interleukin-1 in disease. Blood.

[pmed-0030277-b023] Ware CF (2005). Network communications: Lymphotoxins, LIGHT, and TNF. Annu Rev Immunol.

[pmed-0030277-b024] Nakae S, Naruse-Nakajima C, Sudo K, Horai R, Asano M (2001). IL-1 alpha, but not IL-1 beta, is required for contact-allergen-specific T cell activation during the sensitization phase in contact hypersensitivity. Int Immunol.

[pmed-0030277-b025] Saunders BM, Tran S, Ruls S, Sedgwick JD, Briscoe H (2005). Transmembrane TNF is sufficient to initiate cell migration and granuloma formation and provide acute, but not long-term, control of Mycobacterium tuberculosis infection. J Immunol.

[pmed-0030277-b026] Damon DH, D'Amore PA, Wagner JA (1990). Nerve growth factor and fibroblast growth factor regulate neurite outgrowth and gene expression in PC12 cells via both protein kinase C- and cAMP-independent mechanisms. J Cell Biol.

[pmed-0030277-b027] Means TK, Wang S, Lien E, Yoshimura A, Golenbock DT (1999). Human Toll-like receptors mediate cellular activation by Mycobacterium tuberculosis. J Immunol.

